# Dataset on body weight, carapace width increment and growth band count of mud crabs, *Scylla olivacea*

**DOI:** 10.1016/j.dib.2019.104477

**Published:** 2019-09-04

**Authors:** Mhd Ikhwanuddin, Adnan Amin-Safwan, Nurul Hasyima-Ismail, Mohamad N. Azra

**Affiliations:** a Institute of Tropical Aquaculture and Fisheries Research, Universiti Malaysia Terengganu, 21030, Kuala Nerus, Terengganu, Malaysia; b STU-UMT Shellfish Research Laboratory, Shantou University, Guangdong, 515063, China

**Keywords:** Age determination, Aquaculture, Stock assessment, Immature crabs, Molting, Increment sizes

## Abstract

The present paper contains two datasets; i) the growth band count (GBC) of mud crab, *Scylla olivacea* collected from Setiu Wetlands, Terengganu coastal water, East coast of Peninsular Malaysia and ii) the increment sizes of body weight (BW) and carapace width (CW) of immature *S. olivace* after molting. The datasets presented here were associated with the research articles entitled i) “Study on carapace width growth band counts relationship of orange mud crab, *S. olivacea* (Herbst, 1796) from Terengganu Coastal Waters, Malaysia” (Hasyima-Ismail et al. 2017) [1] and ii) “Relationship between the carapace width and body weight increments and the confirmation of Stage 1 ovary after the molting of immature orange mud crabs, *S. olivacea* (Herbst, 1796), in captivity” (Amin-Safwan et al. 2019-2020) [2], and provided here as raw data of Supplementary materials. Raw datasets for GBC in the wild were generated by examination of the thin cross sectioning process of the gastric mill of *S. olivacea*. The GBC were measured for each individual crab wherein band counts ranged from 1 to 3. The analysis provides evidence that the GBC of the crabs can be determined through both mesocardiac and zygocardiac ossicles. This data is of importance to researchers for estimation of stock assessment and improvement of fisheries management to further improve policy. For the BW-CW increment data, a total of 135 immature crabs were sampled from Setiu Wetlands, Terengganu, Malaysia, and were introduced to limb autotomy technique in order to induced molt. Crabs were reared until successful molting and immediately prior to hardened shell, before final measurement of body weight and carapace width determination. Recorded data was analyzed by calculating the increment sizes, along with correlation and regression analysis between body weight and carapace width of mud crabs.

**Table:** Specifications Table

Subject	Agriculture and Biological Sciences; Aquatic Sciences
Specific subject area	Reproductive biology and age determination
Type of data	Table in the text and Excel in.xlsx
How data were acquired	i) Growth band count: Thin cross sectioning process of gastric mill extraction with longitudinal cutting using a diamond-bladed Isomet Precision Cutter, and ii) Body weight and carapace width increment: Immature crabs were cultured, induced to molt and the data increment of body weight and carapace width were recorded
Data format	Raw and analyzed
Parameters for data collection	i) Growth band count: Only wild mud crab, *Scylla olivacea* with carapace width of > 6 cm were used for the determination of growth band data count (N = 76), and ii) Body weight and carapace width increment: Crabs from the wild were sampled, introduced to limb autotomy procedure, and cultured until completed molting (N = 135)
Description of data collection	i) Growth band count: Examination of mud crab growth band within disarticulated ossicles of the gastric mill which consist of zygocardiac ossicles and mesocardiac ossicles, and ii) Body weight and carapace width increment: Crabs from the wild were sampled, introduced to limb autotomy procedure, and cultured until completed molting before the determination of increment sizes (body weight and carapace width) were recorded for crabs in captive conditions
Data source location	Institute of Tropical Aquaculture and Fisheries Research, Universiti Malaysia Terengganu and Setiu Wetlands Mangrove Forest, Terengganu, Malaysia (5°31′23.1″N 102°55′56.1″E), Wild mud crab samples were collected in the Setiu Wetlands area (latitude: 5˚40ʼ47.93″ N, longitude: 102˚42ʼ45.04″ E)
Data accessibility	With the article
Related research article	*N. Hasyima-Ismail, A. Amin-Safwan, N. Fairuz-Fozi, F.H. Megat, H. Muhd-Farouk, S.A. Kamaruddin, M. Ikhwanuddin, M.A. Ambak. Study on carapace width growth band counts relationship of orange mud crab, Scylla olivacea (Herbst, 1796) from Terengganu Coastal Waters, Malaysia. Pak. J. Biol. Sci. 20 (2017) 140–146.*https://doi.org/10.3923/pjbs.2017.140.146*[1]**and Amin-Safwan, A., Muhd-Farouk, H., Hasyima-Ismail, N., Mahsol, H·H., and Ikhwanuddin, M. Relationship between the Carapace Width and Body Weight Increments and the Confirmation of Stage 1 Ovary after the Molting of Immature Orange Mud Crabs, Scylla olivacea (Herbst, 1796), in captivity. Songklanakarin J. Sci. Tech. “2019–2020”*[*2*]

**Table:** 

**Value of the data** • Absolute age validation and determination using the growth band count would be useful for improvement of fisheries management. • Morphometric analysis such as the length-width relationship could provide a powerful complement to genetic and environmental stock identification approaches, besides being important for population size estimation for fisheries management. • This data can be used to determine the stock assessment plan, connectivity and productivity of different stocks in the wild. • Age information will be useful for future assessment of climate change on the growth of commercially and ecologically important portunid species [3]. • This data can be valuable for joint collaboration with other institutions for establishment of commercialized-research and consultancy unit in the world working on age determination and validation of crustaceans. • Data on weight increment and length-width ratio are widely used in the identification of growth and formation various species in different geographical regions [4], [5].

## 1 Data

The present article contains three types of data, which are available in the form of Supplementary materials GrowthBandDataCount.xlsx (Growth Band Count Data) and Table 1, Table 2, Table 3, Table 4 (Body weight and Carapace Width increment). The GrowthBandDataCount.xlsx data file contains information about raw and mean data of growth band count (GBC), carapace width (CW), and body weight (BW) for both male and female orange mud crab, *S. olivacea* (n = 76). The excel sheet contains analyzed data on the relationship between CW and GBC as well as the frequency of CW between male and female wild mud crab *S. olivacea*. The excel of GrowthBandDataCount.xlsx (as shown in Supplementary Materials) shows male and female Carapace Width (CW), Body Weight (BW) and Growth Band Count (GBC) of known age mud crabs reared in captivity through the zygocardiac ossicles and mesocardiac ossicles of the gastric mill. Table 1, Table 2, Table 3, Table 4 include the raw descriptive data (means), and correlation and regression analysis on the increments of body weight and carapace width relationships of *S. olivacea*. Water parameters were also included such as salinity, temperature, dissolved oxygen and pH and were maintained and monitored daily.

**Table 1:** Carapace width and body weight increment of *S. olivacea* that being introduced with limb autotomized technique. All crabs molted successfully (n = 135).

Carapace Width				Body Weight		
No. of crab	Before (cm)	After (cm)	Size of Increment (cm)	Before (g)	After (g)	Size of Increment (g)
1	7.71	8.91	1.20	103.70	110.50	6.80
2	6.81	7.49	0.68	62.50	69.80	7.30
3	7.11	8.02	0.91	79.10	88.30	9.20
4	7.61	8.65	1.04	78.50	86.70	8.20
5	6.74	7.35	0.61	60.30	68.70	8.40
6	7.74	8.35	0.61	68.90	78.40	9.50
7	8.33	9.37	1.04	86.70	96.40	9.70
8	9.02	10.56	1.54	102.60	110.80	8.20
9	6.92	7.61	0.69	75.60	84.30	8.70
10	7.74	8.44	0.70	80.30	89.70	9.40
11	7.76	8.47	0.71	75.10	83.60	8.50
12	7.04	7.57	0.53	68.30	79.90	11.60
13	8.14	9.33	1.19	98.80	110.50	11.70
14	7.19	7.95	0.76	79.60	86.50	6.90
15	8.14	9.33	1.19	90.50	99.60	9.10
16	7.47	8.17	0.70	79.40	84.60	5.20
17	7.59	8.28	0.69	86.30	94.70	8.40
18	7.39	7.82	0.43	69.60	78.60	9.00
19	6.85	7.59	0.74	68.30	79.40	11.10
20	7.66	8.34	0.68	85.90	98.40	12.50
21	7.86	9.35	1.49	102.20	121.20	19.00
22	6.81	7.13	0.32	67.90	75.40	7.50
23	7.54	8.63	1.09	78.30	84.70	6.40
24	7.17	7.98	0.81	71.90	79.40	7.50
25	6.42	7.56	1.14	57.30	69.90	12.60
26	6.44	7.45	1.01	51.80	63.80	12.00
27	7.95	8.96	1.01	67.70	76.80	9.10
28	6.68	7.32	0.64	55.10	67.90	12.80
29	6.98	7.76	0.78	69.40	80.30	10.90
30	7.17	8.34	1.17	72.90	83.40	10.50
31	6.45	7.38	0.93	52.00	63.70	11.70
32	6.56	7.31	0.75	54.10	63.70	9.60
33	6.19	7.06	0.87	44.30	52.80	8.50
34	7.27	8.07	0.80	77.00	85.70	8.70
35	6.85	7.64	0.79	66.10	75.90	9.80
36	6.59	7.31	0.72	57.10	65.60	8.50
37	7.86	8.54	0.68	63.80	71.50	7.70
38	6.84	7.96	1.12	65.50	76.30	10.80
39	6.88	7.56	0.68	63.40	75.10	11.70
40	6.33	6.92	0.59	51.20	56.50	5.30
41	6.52	7.42	0.90	51.60	57.40	5.80
42	7.61	8.76	1.15	77.30	86.90	9.60
43	7.75	8.41	0.66	95.40	101.20	5.80
44	8.73	9.52	0.79	136.80	148.80	12.00
45	8.76	9.45	0.69	115.80	128.50	12.70
46	8.21	9.18	0.97	109.60	124.30	14.70
47	8.11	8.41	0.30	98.60	101.20	2.60
48	7.12	8.35	1.23	65.40	70.80	5.40
49	8.12	9.52	1.40	139.80	148.80	9.00
50	7.71	8.55	0.84	107.80	111.00	3.20
51	7.81	8.63	0.82	76.90	88.30	11.40
52	7.90	8.01	0.11	68.30	79.70	11.40
53	5.90	6.97	1.07	50.60	60.40	9.80
54	6.81	6.93	0.12	48.60	56.80	8.20
55	8.61	9.66	1.05	114.70	126.50	11.80
56	6.85	7.59	0.74	75.30	84.30	9.00
57	5.95	6.56	0.61	64.20	69.70	5.50
58	9.09	9.79	0.70	88.50	97.80	9.30
59	8.63	9.22	0.59	76.10	85.30	9.20
60	6.26	6.91	0.65	75.40	80.60	5.20
61	7.91	8.52	0.61	73.40	79.60	6.20
62	5.92	6.82	0.90	58.90	64.90	6.00
63	8.28	8.92	0.64	86.70	94.30	7.60
64	5.85	6.63	0.78	63.20	68.50	5.30
65	5.53	6.06	0.53	59.60	68.70	9.10
66	5.45	6.09	0.64	58.50	71.30	12.80
67	9.56	10.24	0.68	91.40	101.3	9.90
68	5.72	6.28	0.56	65.20	71.30	6.10
69	6.52	7.13	0.61	64.10	71.40	7.30
70	6.41	7.14	0.73	64.60	73.10	8.50
71	9.20	9.52	0.32	92.30	95.60	3.30
72	7.07	7.52	0.45	84.10	89.60	5.50
73	6.36	6.63	0.27	64.80	73.80	9.00
74	8.44	8.95	0.51	75.40	81.20	5.80
75	9.15	9.74	0.59	98.60	107.40	8.80
76	7.91	8.84	0.93	69.70	76.30	6.60
77	6.56	7.28	0.72	59.60	71.40	11.80
78	5.85	6.56	0.71	55.80	67.20	11.40
79	8.26	9.02	0.76	75.10	79.50	4.40
80	8.52	9.44	0.92	76.30	88.10	11.80
81	8.15	8.86	0.71	70.50	76.80	6.30
82	7.36	8.55	1.19	66.50	73.30	6.80
83	7.52	7.78	0.26	67.40	76.10	8.70
84	8.48	9.64	1.16	68.90	77.40	8.50
85	7.32	8.27	0.95	57.80	68.50	10.70
86	7.47	8.14	0.67	58.30	64.70	6.40
87	7.82	8.74	0.92	60.30	67.10	6.80
88	7.08	7.69	0.61	56.40	61.70	5.30
89	7.24	8.58	1.34	57.10	61.20	4.10
90	8.15	8.77	0.62	71.50	77.80	6.30
91	8.07	9.21	1.14	65.30	69.80	4.50
92	7.77	8.84	1.07	59.80	64.60	4.80
93	7.33	8.02	0.69	58.20	63.10	4.90
94	6.83	7.85	1.02	55.80	67.20	11.40
95	7.32	8.39	1.07	61.70	68.50	6.80
96	6.76	7.35	0.59	56.90	61.60	4.70
97	7.53	8.42	0.89	60.70	67.90	7.20
98	7.63	8.56	0.93	61.80	68.10	6.30
99	7.49	7.95	0.46	59.60	65.30	5.70
100	7.68	8.09	0.41	61.80	66.70	4.90
101	7.76	8.59	0.83	64.30	71.20	6.90
102	8.58	9.54	0.96	72.40	81.20	8.80
103	7.93	8.82	0.89	65.30	69.60	4.30
104	7.84	8.49	0.65	63.10	69.80	6.70
105	7.14	8.24	1.10	60.80	68.50	7.70
106	7.84	8.98	1.14	72.90	83.60	10.70
107	7.83	8.54	0.71	70.60	79.50	8.90
108	7.93	8.98	1.05	75.40	85.20	9.80
109	7.24	8.36	1.12	68.90	77.90	9.00
110	7.26	7.75	0.49	69.70	76.50	6.80
111	7.74	8.33	0.59	70.80	77.30	6.50
112	7.35	8.07	0.72	69.80	74.30	4.50
113	7.13	7.83	0.70	66.40	75.10	8.70
114	7.68	8.38	0.70	74.30	83.10	8.80
115	7.07	7.73	0.66	66.70	71.80	5.10
116	7.44	8.22	0.78	69.50	76.30	6.80
117	8.44	9.35	0.91	79.60	87.90	8.30
118	8.05	9.26	1.21	77.80	88.40	10.6
119	8.87	9.84	0.97	88.30	92.10	3.80
120	7.16	7.95	0.79	68.30	74.80	6.50
121	6.27	6.65	0.38	59.70	68.30	8.60
122	6.63	7.39	0.76	68.10	78.90	10.8
123	6.56	7.29	0.73	67.10	71.70	4.60
124	7.86	8.48	0.62	75.90	79.90	4.00
125	8.26	9.43	1.17	69.60	78.90	9.30
126	7.69	8.66	0.97	73.20	84.30	11.10
127	7.84	8.78	0.94	68.30	76.90	8.60
128	8.05	8.68	0.63	73.20	85.10	11.90
129	8.39	9.44	1.05	72.40	83.30	10.90
130	7.61	8.61	1.00	66.70	78.10	11.40
131	7.62	8.55	0.93	68.90	76.30	7.40
132	8.90	9.90	1.00	76.90	89.30	12.40
133	8.35	9.44	1.09	63.20	75.10	11.90
134	7.43	8.82	1.39	65.20	75.10	9.90
135	8.67	10.17	1.50	77.80	89.20	11.40

**Table 2:** Mean, standard deviation, highest and lowest carapace width and body weight increments recorded after the crabs molted (n = 135).

	Mean	Standard deviation	Highest increment	Lowest increment
CW (cm)	0.816	0.27	1.54	0.30
BW (g)	8.395	2.72	19.00	3.20

**Table 3:** Correlation analysis for carapace width and body weight increments of *S. olivacea* (n = 135).

		CW	BW
CW	Pearson Correlation	1	0.263**
	Sig. (1-tailed)		0.001
	N	135	135

**Table 4:** Regression analysis for carapace width and body weight increments of *S. olivacea* (n = 135).

Model		Sum of Squares	df	Mean Square	F	Sig.
1	Regression	68.665	1	68.665	9.897	0.002^b^
	Residual	922.702	133	6.938		
	Total	991.366	134			

### 2.1 Experimental design, materials, and methods

#### 2.1.1 Growth band count in the wild

##### 2.1.1.1 Crab collection

Male and female crab specimens were collected from Setiu Wetlands, Terengganu, Malaysia. Samplings were carried out from February to August 2016. Conventional circular-shape folding crab pots (mesh size of 1 cm and diameter of 40 cm) with trash fish bait were used to capture the crabs [6], [7]. Seventy-six crabs (31 females and 45 males) were collected and their CW and BW were measured per sampling sites. Crabs were managed, identified and sexed based on the following existing criteria [8], [9]. All crabs were labelled using cable tie tags then their cardiac stomachs were exposed to extract the gastric mill.

##### 2.1.1.2 Growth band counts (GBC) analysis

All of the gastric mill samples were preserved in a 4% glycerol, 26% water and 70% ethanol solution for subsequent GBC analysis. After four days, the gastric mill were separated between mesocardiac ossicles and zygocardiac ossicles, cleaned from excess tissues and embedded in Buehler epoxy resin. Both ossicles were then prepared for transverse sectioning between 150 and 300 μm thicknesses using Buehler Isomet Precision Saw [10], [11]. Both ossicles were mounted on slides and viewed under an Olympus microscope, while images were taken using DinoCapture software and digitally enhanced using Adobe Photoshope. Data of GBC were based on the identification of paired (bipartite) light and dark zones in the endocuticle and counted from basal (membranous layer and hypodermis) to distal region of the endocuticle of the structure [10], [11].

#### 2.1.2 Body weight and carapace width increment

##### 2.1.2.1 Sampling and crab management

A total of 135 immature female *S. olivacea* (carapace width, CW less than 9.06 cm; small and pale abdominal flap) were sampled from Setiu Wetlands, Terengganu, East Coast of Peninsular Malaysia (5° 31′23.1″N 102° 55′56.1″E). Conventional rectangular collapse crab pots (dimension: length x width x height = 87 cm × 56 cm x 30 cm; mesh size = 1 cm) with openings at the middle of both end sections were used during sampling section [12]. Crab pots were deployed during the low tide in the evening (1600 h) and collected during subsequent low tide in the morning of the next day (0800 h). Only immature female *S. olivacea* were chosen, and then the crab samples were transferred to the Institute of Tropical Aquaculture and Fisheries Research Marine Hatchery, Universiti Malaysia Terengganu for subsequent analysis.

The initial body weight and carapace width of each crab were measured and recorded. Body weight was measured using a digital balance (accuracy: 0.01 g; Shimadzu model, Japan), whereas carapace width, the distance between the tips of the 9th anterolateral spine of the crab carapace [13], [14] was determined using a six-inch liquid crystal display (LCD) digital Vernier caliper (accuracy: 0.01 cm; Kingsmart brand, Hong Kong). The crabs were then introduced to the limb autotomy procedure, placed individually in a container, fed with chopped Scadfish, *Selaroides leptolepis*, at 10% of their body weight twice daily (at 0900 and 1700 h), and were maintained until molting was completed. The final measurement of body weight and carapace width was measured once a crab successful molted, and their shells completely hardened (approximately ±7 days after molting event).

##### 2.1.2.2 Limb autotomy

The chelipeds (claws) and pereiopods (walking legs) were cast off, leaving only the pleopods (swimming legs) for the crabs’ movement [15]. The autotomized crabs were placed individually in a container, with ambient salinity (28–32 ppt), maintained temperature (27–29 °C), moderate aeration, ambient light intensity, and 100% water exchange every two days. The crabs were fed with chopped *S. leptolepis*, at 10% of their body weight twice daily (at 0900 and 1700 h) for observation of the molting event.

##### 2.1.2.3 Data analysis

The body weight and carapace width increment sizes were measured and recorded. The incrementation was calculated as follows:

Size of increment = Final body weight/carapace width – Initial body weight/carapace width.

The collected data were analyzed using statistical correlation and regression analysis through the Statistical Package for the Social Science (SPS) software (Version 22.0 for Windows; SPSS Inc. Armonk, NY: IBM Corp.), available at https://www.ibm.com/products/spss-statistics.
